# Prostate cancer‐related anxiety among long‐term survivors after radical prostatectomy: A longitudinal study

**DOI:** 10.1002/cam4.5304

**Published:** 2022-10-18

**Authors:** Valentin H. Meissner, Cornelia Peter, Donna P. Ankerst, Stefan Schiele, Jürgen E. Gschwend, Kathleen Herkommer, Andreas Dinkel

**Affiliations:** ^1^ Department of Urology Klinikum rechts der Isar, School of Medicine, Technical University of Munich Munich Germany; ^2^ Departments of Mathematics and Life Science Systems Munich Data Science Institute, Technical University of Munich Garching Germany; ^3^ Department of Psychosomatic Medicine and Psychotherapy Klinikum rechts der Isar, School of Medicine, Technical University of Munich Munich Germany

**Keywords:** anxiety, prostatectomy, prostatic neoplasms, quality of life, survivorship

## Abstract

**Background:**

Prostate cancer (PC)‐related anxiety is associated with clinically significant declines in health‐related quality of life (HRQoL) and psychological well‐being. This longitudinal study investigates course and predictors of PC‐related anxiety in long‐term PC survivors treated by radical prostatectomy (RP).

**Methods:**

Two thousand nine hundred and three survivors from the multicenter German Familial PC Database completed the Memorial Anxiety Scale for PC on average 11 years after RP at the initial assessment in 2015 and then 5 years later. Hierarchical multiple linear regression was used to assess predictors of PC‐related anxiety at follow‐up.

**Results:**

PC‐related anxiety remained stable over the 5 years. In hierarchical multiple linear regression, longitudinal predictors of PC‐related anxiety 5 years later included a lower level of education (beta: −0.035, *p* = 0.019), biochemical recurrence (BCR; beta: 0.054, *p* = 0.002), late BCR (beta: 0.054, *p* < 0.001), PC anxiety at initial assessment (beta: 0.556, *p* < 0.001), HRQoL (beta: −0.076, *p* < 0.001), depression and anxiety symptoms (beta: 0.072, *p* = 0.001; beta: 0.165, *p* < 0.001). Predictors of prostate‐specific antigen (PSA) anxiety 5 years later included late BCR (beta: 0.044, *p* = 0.019), PSA anxiety at initial assessment (beta: 0.339, *p* < 0.001), depression and anxiety symptoms (beta: 0.074, *p* = 0.008; beta: 0.191, *p* < 0.001), and treatment decision regret (beta: 0.052, *p* = 0.006).

**Conclusion:**

PC‐related anxiety remains a burden to survivors many years after diagnosis and treatment. The respective disease‐specific anxiety was the strongest predictor of this anxiety 5 years later, which emphasizes the need of screening and monitoring in a timely manner for PC‐related anxiety. Treating urologists should screen, identify, and monitor patients at risk for targeted referrals to psychosocial services.

## Graphical abstract

Findings of the current study highlight PC‐related anxiety as a burden of survivors many years after diagnosis and treatment. The respective disease‐specific anxiety was the strongest predictor of this anxiety 5 years later, which emphasizes the need of screening and monitoring in a timely manner for PC‐related anxiety. Treating urologists should screen, identify, and monitor patients at risk for targeted referrals to psychosocial services.

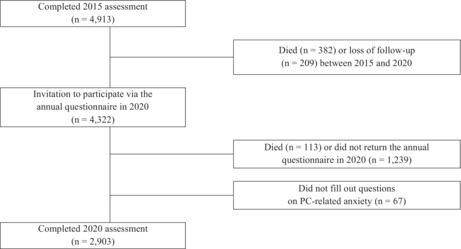

## 1 INTRODUCTION

Currently approximately 6 million prostate cancer (PC) survivors live in the United States and in Europe.^1^, ^2^ Given that long‐term survival is common after definitive treatment, addressing survivors' needs and improvements in quality of life are integral to post‐treatment clinical care. In addition to somatic effects, disease‐related anxiety, such as that related to prostate‐specific antigen (PSA) testing, is associated with clinically significant declines in health‐related quality of life (HRQoL) and psychological well‐being.^3^, ^4^, ^5^, ^6^ The American Cancer Society Prostate Cancer Survivorship Care Guidelines state that “survivors with significant or persistent PSA anxiety may be at heightened risk of depressive symptoms or general distress” and recommend regular screening and follow‐up.^7^ To improve recognition and identification of anxiety related to PC, Roth et al. developed the Memorial Anxiety Scale for Prostate Cancer (MAX‐PC), which assesses anxiety related to PC in general, PSA testing, and fear of recurrence.^8^

To date, previous research on PC‐related anxiety has primarily focused on active surveillance^9^, ^10^ or short‐term post‐treatment care.^5^, ^11^ Evidence is limited for long‐term effects among PC survivors. Previous results of our cross‐sectional study of German PC survivors showed significant PC‐related anxiety among some men more than 10 years after diagnosis and treatment.^12^ Longitudinal data on the further course of PC‐related anxiety assessing risk factors in long‐term survivors will help clinicians to improve survivorship care and to identify survivors at risk to provide appropriate psychological care when needed. A recently published systematic review highlighted fear of cancer recurrence and PSA anxiety as important symptoms associated with poorer HRQoL and mental health status. Therefore, the authors recommended screening for these constructs and referral to appropriate services as part of routine follow‐up care.^13^

The objectives of the current study were to (1) assess the prevalence of longitudinal PC‐related anxiety in long‐term survivors over a 5‐year period and (2) identify and assess predictors of PC‐related anxiety 5 years after initial assessment in long‐term survivors after radical prostatectomy (RP) of a large registry‐based national sample.

## 2 METHODS

### 2.1 Study procedure and patient population

This study was approved by the Technical University of Munich ethical review committee, with written informed consent obtained from all participants. PC survivors for this study were recruited among the 40,000 patients and relatives participating in the multi‐center German Familial Prostate Cancer prospective study, which has surveyed newly PC diagnosed patients with follow‐up annual questionnaires since 1994. Detailed descriptions of study methodology have been previously reported.^14^

**FIGURE 1:**
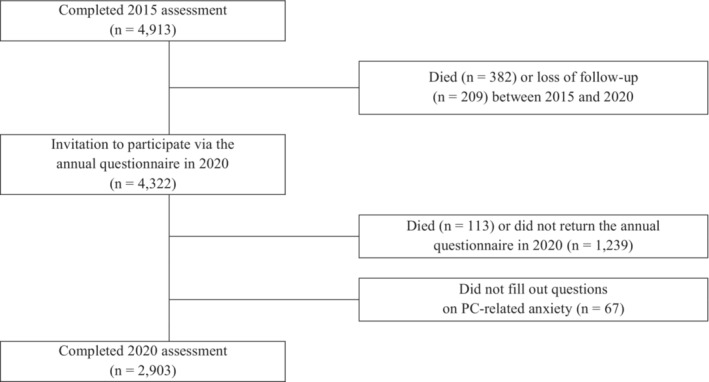
Flowchart of study design and number of patients.

Patients with histologically proven PC treated with RP as first‐line treatment who submitted questionnaires of PC‐related anxiety at initial assessment in October 2015 and at follow‐up in October 2020 were included in the study (Figure 1). A dropout analysis in 2020 showed that the 1419 patients who did not return the annual questionnaire (*n* = 1239), died (*n* = 113) or did not fill out questions on PC‐related anxiety (*n* = 67) were older at survey in 2015 (*M* = 76.4 vs. *M* = 73.8 years; *p* < 0.001), less educated (*p* < 0.001), had more often a biochemical recurrence (BCR; 36.2% vs. 30.4%; *p* < 0.001), lower HRQoL (*M* = 70.5 vs. *M* = 74.7; *p* < 0.001), and had more often depressive (10.9% vs. 7.2%; *p* < 0.001) and anxiety (8.4% vs. 6.7%; *p* = 0.049) symptoms in comparison with included patients (*n* = 2903). PC and PSA anxiety assessed with the MAX‐PC in 2015 did not differ (*p* = 0.579; *p* = 0.243) (Figure 1).

### 2.2 Measures

The following measures were assessed in 2015 and 2020. PC‐related anxiety was assessed using the MAX‐PC, a validated and reliable 18 item instrument that measures overall anxiety related to PC in general, PSA testing, and fear of recurrence in three subscales.^8^, ^15^ To reduce participant burden, a modified PC anxiety subscale with 4 of the original 11 items and all 3 items of the PSA anxiety subscale were selected. Responses were made on a four‐point Likert scale (0 = not at all to 3 = often). This yielded minimum scores of 0 and maximum scores of 12 for the PC anxiety scale and maximum scores of 9 for the PSA anxiety subscale, respectively, with higher scores indicating more anxiety. Cronbach's alpha coefficients in the current sample were *α* = 0.88 for both 2015 and 2020 for the PC anxiety subscale, and 0.73 and 0.76 for the PSA anxiety subscale, in 2015 and 2020, respectively.

Sociodemographic characteristics included age at survey, level of education (low, intermediate, high, tertiary), partnership, and children. Clinicopathological characteristics included years since RP, positive PC family history (defined as at least one relative with PC who did not die of PC), lethal PC family history (at least one first‐degree relative who died of PC), secondary cancer, PSA at diagnosis, organ‐defined disease at RP, BCR defined as a PSA level ≥0.2 ng/ml between RP and 2015, late BCR (between 2015 and 2020), and current therapy (radiation therapy, androgen deprivation, and chemotherapy vs. none).

Health‐related quality of life was assessed using items 29 and 30 of the European Organization for Research and Treatment of Cancer QLQ‐C30.^16^ Calculated mean scores were transformed to a range between 0 and 100, a higher score represents better HRQoL. Cronbach's alpha was *α* = 0.90 and 0.91 in 2015 and 2020, respectively. Depression and anxiety symptoms were assessed using the Patient Health Questionnaire‐2 and the Generalized Anxiety Disorder scale‐2, respectively. Items are scored from 0 to 3. For both measures, a summary score ≥3 represents a cut‐off indicating clinical levels of depression and anxiety, respectively.^17^ Cronbach's alpha coefficients were *α* = 0.70 for the depression scale in both 2015 and 2020, and 0.72 and 0.77, respectively, for the anxiety scale.

Decision regret related to the initial PC treatment decision of RP was assessed retrospectively in 2020, using one item from the Decision Regret Scale.^18^ Patients were asked whether *they would go for the same choice if they had to do it over again (yes* vs. *no)*. This item was chosen because it demonstrated the highest item‐total correlation in a previous study of breast cancer surgery and PC treatment.^18^

### 2.3 Statistical analysis

Descriptive statistics calculating counts and percentages for categorical variables and means and standard deviations (SD) for continuous variables were used to present participant characteristics in 2015 and 2020. Differences in the frequency of responses to the MAX‐PC items between 2015 and 2020 were calculated using the McNemar‐Bowker test. Hierarchical multiple linear regression analysis was applied to identify and assess predictors of PC‐related anxiety in 2020 via characteristics available at 2015 (step 1) and characteristics available at 2020 (step 2). Results were reported in terms of linear slopes, corresponding to the change in anxiety score (with minimum 0 and maximum 12 and 9 points, respectively, and higher scores worse) corresponding to a one‐unit increase in continuous variables, and by factor level for categorical variables. For example, a slope of 0.5 for the predictor age at survey indicates that the total PC anxiety score increased on average by 0.5 points for an increase of 1 year in age and a slope of 0.5 for the predictor HRQoL indicates that the total PC anxiety score increased on average by 0.5 points for an increase of 10 points. All tests were two‐sided and *p*‐values <0.05 were considered statistically significant. All analyses were performed using SAS 9.4.

## 3 RESULTS

### 3.1 Characteristics of the study population

Table 1 shows characteristics of the study population of 2903 former RP patients in 2015 and 2020. Mean age at the initial assessment in 2015 was 73.8 (SD 6.3) years and mean time since RP was 11.5 (SD 3.7) years. Mean HRQoL declined from 74.7 (SD 17.1) to 69.8 (SD 19.2) during the five‐year span (*p* < 0.001). According to published reference data, this change can be considered a small deterioration.^19^ Prevalence of clinical levels of depression at the initial assessment and 5 years later were 7.2% and 8.5% (*p* = 0.066), respectively, and of anxiety 6.6% and 7.0% (*p* = 0.332), respectively. Approximately 16 years after RP, 10.8% expressed treatment decision regret (Table 1).

**TABLE 1:** Patient characteristics of the study population (*N* = 2903)

	2015	2020
Sociodemographic characteristics		
Age at survey, mean (SD), years	73.8 (6.3)	78.8 (6.3)
Age at survey, median (IQR), years	74.8 (70.3–78.1)	79.8 (75.3–83.1)
Level of education, No. (%)		
Low	1104 (39.1)	
Intermediate	507 (18.0)	
High	353 (12.5)	
Tertiary	859 (30.4)	
Partnership, No. (%)	2607 (93.1)	
Children, No. (%)	2564 (88.7)	
Clinicopathological characteristics		
Years since RP, mean (SD), years	11.5 (3.7)	16.5 (3.7)
Years since RP, median (IQR), years	11.1 (9.1–13.9)	16.1 (14.1–18.9)
Positive PC family history, No. (%)	891 (30.7)	
Lethal PC family history, No. (%)	287 (9.9)	
Secondary cancer, No. (%)	312 (10.8)	
Secondary cancer between 2015 and 2020, No. (%)		35 (1.2)
PSA at diagnosis, median (IQR), ng/ml	7.2 (5.2–11.0)	
Organ defined disease at RP, No. (%)	2048 (71.1)	
BCR between RP and 2015, No. (%)	882 (30.4)	
BCR between 2015 and 2020, No. (%)		167 (5.8)
Current therapy, No. (%)	339 (11.7)	321 (11.1)
HRQoL and psychosocial characteristics		
HRQoL, mean (SD)	74.7 (17.1)	69.8 (19.2)
HRQoL, median (IQR)	83.3 (66.7–83.3)	75.0 (58.3–83.3)
Depression, No. (%)	207 (7.2)	237 (8.5)
Anxiety, No. (%)	190 (6.6)	196 (7.0)
Decision regret, No. (%)		304 (10.8)

*Note*: The numbers indicated are among the completed entries and not always adding up to the total sample size.

Abbreviations: BCR, biochemical recurrence; HRQoL, health‐related quality of life; IQR, interquartile range; PC, prostate cancer; PSA, prostate‐specific antigen; RP, radical prostatectomy; SD, standard deviation.

The frequency of responses to the items of the MAX‐PC are displayed in Table 2. Response options “sometimes” and “often” were merged into the same category due to the infrequent choice of the maximum score, following our previous publication of the cross‐sectional analysis from 2015.^12^ Prevalence of significant PC‐related anxiety (i.e., sometimes/often) remained stable or slightly increased over the 5 years. PC anxiety varied between 13% and 24% at both assessments (2015 and 2020), whereas prevalence of PSA anxiety was low (about 3%) at both assessments (Table 2).

**TABLE 2:** Frequency of response to the items of the PSA anxiety subscale and the modified PC anxiety subscale of the MAX‐PC

		*n* (%) in 2015	*n* (%) in 2020	*p* Value
PC anxiety subscale				
Strong feelings	Not at all	1364 (47.3)	1269 (44.5)	
	Rarely	909 (31.5)	895 (31.4)	0.001
	Sometimes/often	609 (21.1)	689 (24.1)	
Scared of PSA test	Not at all	1805 (62.7)	1673 (58.4)	
	Rarely	572 (19.9)	647 (22.6)	<0.001
	Sometimes/often	503 (17.4)	546 (19.1)	
Conferred anxiety	Not at all	1830 (63.4)	1812 (63.2)	
	Rarely	632 (21.9)	676 (23.6)	0.160
	Sometimes/often	423 (14.7)	380 (13.3)	
Anxiety before PSA test	Not at all	1775 (61.6)	1744 (61.0)	
	Rarely	629 (21.8)	619 (21.7)	0.263
	Sometimes/often	478 (16.6)	495 (17.3)	
PSA anxiety subscale				
Delaying PSA test	Not at all	2653 (91.9)	2598 (90.2)	
	Rarely	155 (5.4)	186 (6.5)	0.049
	Sometimes/often	80 (2.7)	96 (3.3)	
Repeat PSA test	Not at all	2605 (90.4)	2518 (87.6)	
	Rarely	188 (6.5)	252 (8.8)	<0.001
	Sometimes/often	90 (3.1)	103 (3.6)	
PSA test elsewhere	Not at all	2673 (92.8)	2642 (92.2)	
	Rarely	125 (4.3)	153 (5.3)	0.317
	Sometimes/often	83 (2.9)	71 (2.5)	

Abbreviations: MAX‐PC, Memorial Anxiety Scale for Prostate Cancer; PC, prostate cancer; PSA, prostate‐specific antigen.

### 3.2 Hierarchical multiple linear regression analysis

In the multiple linear regression model for PC anxiety in 2020 adjusting for risk factors in 2015, a lower level of education remained a predictor for high PC anxiety (standardized regression coefficient beta: −0.035; *p* = 0.019). BCR between RP and 2015 as well as late BCR between 2015 and 2020 was associated with higher levels of PC anxiety in 2020 (beta: 0.054; *p* = 0.002 and beta: 0.054; *p* < 0.001, respectively). High PC anxiety at initial assessment was the strongest predictor of PC anxiety in 2020 (beta: 0.556; *p* < 0.001). Both depression and anxiety symptoms in 2020 were associated with higher PC anxiety in 2020 (beta: 0.072; *p* = 0.001, beta: 0.165; *p* < 0.001, respectively) and lower HRQoL in 2020 was associated with higher PC anxiety (beta: −0.076; *p* < 0.001) (Table 3).

**TABLE 3:** Hierarchical multiple linear regression analysis, variables regressed on PC anxiety in 2020, adjusting for risk factors in 2015

	Step 1				Step 2			
	*B*	SE *B*	Beta	*p* Value	*B*	SE *B*	Beta	*p* Value
2015 risk factors								
Age at survey^a^	0.001	0.008	0.001	0.957	−0.006	0.008	−0.012	0.460
Level of education (low = 0)	−0.110	0.036	−0.048	0.003	−0.081	0.035	−0.035	0.019
Partnership (no = 0)	0.213	0.186	0.018	0.252	0.231	0.177	0.020	0.193
Children (no = 0)	−0.156	0.145	−0.017	0.281	−0.106	0.138	−0.012	0.444
Years since RP^a^	−0.003	0.013	−0.003	0.845	−0.005	0.013	−0.006	0.690
Lethal PC family history (no = 0)	−0.217	0.158	−0.022	0.168	−0.239	0.150	−0.024	0.112
Positive PC family history (no = 0)	−0.021	0.101	−0.003	0.832	−0.013	0.097	−0.002	0.893
Secondary cancer (no = 0)	0.109	0.144	0.012	0.448	−0.068	0.138	−0.007	0.623
BCR (no = 0)	0.348	0.112	0.055	0.002	0.341	0.112	0.054	0.002
Current therapy (no = 0)	0.154	0.160	0.017	0.335	0.125	0.184	0.013	0.499
PC anxiety^a^	0.597	0.018	0.593	<0.001	0.560	0.017	0.556	<0.001
HRQoL^a^	−0.040	0.032	−0.023	0.209	0.052	0.033	0.030	0.119
Depression^a^	0.074	0.059	0.027	0.209	−0.022	0.058	−0.008	0.699
Anxiety^a^	0.191	0.060	0.068	0.002	−0.030	0.060	−0.011	0.616
2020 risk factors								
Secondary cancer since 2015 (no = 0)					−0.338	0.385	−0.013	0.380
BCR since 2015 (no = 0)					0.680	0.190	0.054	<0.001
Current therapy (no = 0)					−0.047	0.191	−0.005	0.806
HRQoL^a^					−0.118	0.031	−0.076	<0.001
Depression^a^					0.186	0.058	0.072	0.001
Anxiety^a^					0.433	0.059	0.165	<0.001
Decision regret (no = 0)					0.249	0.149	0.026	0.086

*Note*: Adjusted *R* ^2^ = 0.492.

Abbreviations: B, unstandardized regression coefficient; BCR, biochemical recurrence; beta, standardized regression coefficient; HRQoL, health‐related quality of life; PC, prostate cancer; RP, radical prostatectomy; SE, standard error.

^a^ Variables treated as continuous.

In the multiple linear regression model for PSA anxiety in 2020 adjusting for risk factors in 2015, high PSA anxiety at initial assessment was the strongest predictor of PSA anxiety in 2020 (beta: 0.339; *p* < 0.001). BCR between RP and 2015 was not associated with increased PSA anxiety, but late BCR between 2015 and 2020 was associated with higher PSA anxiety (beta: 0.013; *p* = 0.553 and beta: 0.044; *p* = 0.019, respectively). Both depression and anxiety symptoms in 2020 were associated with higher PSA anxiety (beta: 0.074; *p* = 0.008, beta: 0.191; *p* < 0.001, respectively). Treatment decision regret in 2020 was associated with higher PSA anxiety (beta: 0.052; *p* = 0.006) (Table 4).

**TABLE 4:** Hierarchical multiple linear regression analysis, variables regressed on PSA anxiety in 2020, adjusting for risk factors in 2015.

	Step 1				Step 2			
	*B*	SE *B*	Beta	*p* Value	*B*	SE *B*	Beta	*p* Value
2015 risk factors								
Age at survey^a^	−0.001	0.003	−0.005	0.801	−0.002	0.003	−0.010	0.624
Level of education (low = 0)	−0.022	0.016	−0.027	0.167	−0.010	0.016	−0.012	0.530
Partnership (no = 0)	0.012	0.082	0.003	0.882	0.010	0.080	0.002	0.897
Children (no = 0)	0.011	0.064	0.003	0.866	0.031	0.062	0.009	0.617
Years since RP^a^	−0.007	0.006	−0.025	0.221	−0.008	0.006	−0.026	0.193
Lethal PC family history (no = 0)	−0.006	0.070	−0.002	0.930	−0.009	0.068	−0.002	0.897
Positive PC family history (no = 0)	−0.002	0.045	−0.001	0.956	0.003	0.044	0.001	0.951
Secondary cancer (no = 0)	−0.057	0.064	−0.017	0.367	−0.115	0.062	−0.034	0.063
BCR (no = 0)	0.032	0.049	0.014	0.509	0.030	0.050	0.013	0.553
Current therapy (no = 0)	−0.087	0.071	−0.026	0.221	−0.086	0.083	−0.026	0.302
PSA anxiety^a^	0.372	0.021	0.358	<0.001	0.352	0.020	0.339	<0.001
HRQoL^a^	−0.020	0.014	−0.032	0.153	0.001	0.015	0.002	0.939
Depression^a^	0.036	0.026	0.037	0.170	−0.004	0.026	−0.004	0.889
Anxiety^a^	0.067	0.026	0.066	0.011	−0.023	0.027	−0.023	0.387
2020 risk factors								
Secondary cancer since 2015 (no = 0)					−0.047	0.174	−0.005	0.786
BCR since 2015 (no = 0)					0.201	0.086	0.044	0.019
Current therapy (no = 0)					−0.057	0.086	−0.017	0.508
HRQoL^a^					−0.009	0.014	−0.016	0.530
Depression^a^					0.069	0.026	0.074	0.008
Anxiety^a^					0.181	0.026	0.191	<0.001
Decision regret (no = 0)					0.181	0.065	0.052	0.006

*Note*: Adjusted *R* ^2^ = 0.212.

Abbreviations: B, unstandardized regression coefficient; BCR, biochemical recurrence; beta, standardized regression coefficient; HRQoL, health‐related quality of life; PC, prostate cancer; PSA, prostate‐specific antigen; RP, radical prostatectomy; SE, standard error.

^a^ Variables treated as continuous.

## 4 DISCUSSION

This study showed that even after a median follow‐up of 16.5 years, significant levels of PC‐related anxiety were still present in a notable number of PC survivors.

The strongest predictors for both PC and PSA anxiety were the respective anxiety scores 5 years previous, underscoring the persistence of post‐RP anxiety. Clinicians and care‐givers should monitor disease‐specific anxieties early after treatment to identify patients at risk in a timely manner and initiate psychological interventions when needed. Recently published results from our group about fear of cancer recurrence in German long‐term PC survivors showed comparable results.^20^ Fear of cancer recurrence, which is likewise a disease‐related anxiety and stems from the real threat of cancer returning of progressing, was persistent even many years after diagnosis and treatment. High levels of fear of cancer recurrence were associated with a 10‐fold increase in the odds of having fear of cancer recurrence about 10 years later. This underlines the clinically relevant persistence of this anxiety and the need of an early identification of patients at risk. In this analysis, similar factors such as BCR or anxiety and depression symptoms showed associations with increased levels of fear of cancer recurrence. However, other factors such as current PC therapy or years since RP were associated with higher levels of fear of cancer recurrence but were not related to PC or PSA anxiety in the current analysis, which suggests that there are some differences in these disease‐related anxiety.

Prostate‐specific antigen testing is a fundamental part of clinical PC follow‐up care since PSA is a reliable marker of disease recurrence.^21^ It is known that periodic PSA testing post therapy is associated with increased levels of anxiety with almost one third of survivors experiencing anxiety before testing.^22^ This anxiety specifically related to PSA testing can lead to delayed testing or requests of repeat tests to ensure accuracy, which may interfere with effective disease management.^8^ Results of the current study indicated a low level of PSA anxiety with about 3% of survivors experiencing significant levels of anxiety related to PSA testing at the initial assessment in 2015, though this rate remained stable at follow‐up. While previous studies reported likewise low item endorsement of the PSA anxiety subscale,^15^, ^23^ collected prevalence rates of PSA anxiety must be interpreted with caution, since anxiety related to PSA testing might not be experienced constantly and might be triggered by follow‐up appointments or environmental triggers (i.e., internet, news, TV). Since annual follow‐up questionnaires in this study were sent independently of scheduled follow‐up care appointments, results may not have detected high levels of PSA anxiety that men experienced shortly before PSA testing.

Anxiety related to PC in general showed a notable prevalence of 15%–21% at initial assessment and remained stable or slightly increased in some items 5 years later. Taking the long follow‐up period of 11.5 and 16.5 years, respectively, into account, it is noteworthy that PC anxiety remains stable in certain survivors despite the good prognosis and the low rates of late BCR.^24^ This high prevalence and furthermore, the association with decreased HRQoL emphasize the need of an early identification of patients at risk to make timely and appropriate referrals to psycho‐oncologists. A systematic review has recently highlighted the utility of psychological interventions for PC‐related depression, anxiety, and distress.^25^

A secondary cancer, a positive family history of PCa as well as having a relative who died of PC were all unrelated with PC‐related anxiety among PCa survivors of the current analysis. These findings are in line with previous studies.^10^, ^11^, ^26^ Although a familial predisposition is a well‐recognized risk factor of PC, it is not associated with worse long‐term outcomes of PCa survivors.^27^

Higher depression and anxiety scores at follow‐up in 2020 were both associated with higher PC‐related anxiety, which has likewise been described in the literature.^5^, ^26^ However, the causal direction of the relationship is unknown. Further psychological factors such as intolerance of uncertainty or productivity loss are described to be associated with PC‐related anxiety.^5^, ^26^ These results suggest that survivors with a reduced psychological well‐being are at increased risk of experiencing PC‐related anxiety.

Late BCR (i.e., between the initial assessment and follow‐up) was associated with higher PC and PSA anxiety. Interestingly, an earlier BCR (i.e., between RP and initial assessment) was only associated with PC anxiety, but not with PSA anxiety. Apparently, having had BCR with a presumable consecutive and successful treatment many years before might lead to certainty about the follow‐up management including PSA testing with reduced anxiety and distrust related to PSA testing.

Results of the current study indicated that about 11% of PC survivors expressed treatment decision regret, which is in line with previous results.^28^ While PC anxiety was not associated with decision regret, anxiety related to PSA testing showed an association with decision regret. Besides functional and oncologic outcomes,^29^ higher PSA concern has likewise been reported to be associated with decision regret in long‐term PC survivors.^28^

Previous studies have shown that younger age is associated with higher levels of PC‐related anxiety.^8^, ^11^, ^30^ Likewise, results of our previously published cross‐sectional analysis from 2015 showed an association between a younger age and higher risk of PC‐related anxiety.^12^ However, all these studies are limited by their cross‐sectional design, whereas the current multivariable regression analyses assessed longitudinally the trajectory of PC‐related anxiety. In fact, when assessing cross‐sectionally PC‐related anxiety in 2020 in the current sample (i.e., without including PC‐related anxiety of 2015), younger age is also associated with higher levels of PC‐related anxiety (*B*: ‐0.047, SE *B*: 0.009, beta: −0.100, *p* < 0.001) (Data not shown). However, after adjusting for PC‐related anxiety in 2015 in the multivariable regression analyses, age is not associated anymore with PC‐related anxiety in 2020, which means that there is no additional effect of age as a predictor for longitudinal PC‐related anxiety. Notably, median age at survey of our sample of PC survivors was 74.8 years (in 2015) and 79.8 years (in 2020), respectively. Therefore, results of our analyses are limited to PC survivors at this age and are not automatically transferable to younger PC survivors. Since aforementioned studies investigated mostly younger men (Roth et al. assessed men with a median age of 71 years^8^; Tavlarides et al. with a median age of 64 years^11^; Lintz et al. with a median age of 70 years^30^), there might be an effect of age in younger PC survivors.

To date, the current study has been the largest registry study which assessed prevalence and predictors of PC‐related anxiety longitudinally in long‐term PC survivors after RP. However, some limitations should be considered when interpreting its findings. Data were derived from a RP cohort so that results are not representative of all PCa survivors. Most data are self‐reported and at risk for under or overstatement. Results of the dropout analysis in 2020 showed that non‐respondents were older, had more often a BCR, and reported poorer emotional health (HRQoL, depressive/anxiety symptoms). These non‐respondents could contribute to non‐random missing data resulting in a higher risk of bias. Lastly, the current study was performed in Germany and several cultural differences in coping or health care utilization might exist compared to PC survivors from other parts of the world. However, there is currently no evidence in the literature that this could affect results obtained in a significant manner.

## 5 CONCLUSIONS

This study underlines PC‐related anxiety as a burden that is still present in some PC survivors even many years after treatment. The respective disease‐specific anxiety was the strongest predictor of this anxiety 5 years later, which emphasizes the need of screening and monitoring in a timely manner for PC‐related anxiety. Identification of patients at risk enables initiation of early psychological interventions, which has been shown to be an effective treatment strategy. Lower level of education, BCR, depressive and anxiety symptoms, decreased HRQoL, and decisional regret were further important predictors of PC‐related anxiety in survivors. Therefore, treating urologists should be aware of these factors in clinical practice to make timely and appropriate referrals to psycho‐oncologists, as disease‐related anxieties are leading to limitations in HRQoL and psychological well‐being.

## AUTHOR CONTRIBUTIONS

**Valentin H. Meissner:** Conceptualization (lead); data curation (supporting); formal analysis (equal); investigation (equal); methodology (equal); software (equal); validation (equal); writing – original draft (lead); writing – review and editing (lead). **Cornelia Peter:** Data curation (lead); writing – review and editing (supporting). **Donna P. Ankerst:** Formal analysis (equal); methodology (equal); validation (equal); writing – review and editing (equal). **Stefan Schiele:** Formal analysis (lead); methodology (equal); software (lead); writing – review and editing (equal). **Jürgen E. Gschwend:** Project administration (equal); supervision (equal); writing – review and editing (equal). **Kathleen Herkommer:** Conceptualization (equal); data curation (equal); project administration (lead); supervision (lead); validation (equal); writing – review and editing (equal). **Andreas Dinkel:** Conceptualization (lead); formal analysis (equal); methodology (equal); supervision (equal); validation (equal); writing – review and editing (equal).

## CONFLICT OF INTEREST

The authors have no relevant financial or non‐financial interests to disclose.

## ETHICAL APPROVAL STATEMENT

All procedures performed in studies involving human participants were in accordance with the ethical standards of the institutional and/or national research committee and with the 1964 Helsinki Declaration and its later amendments or comparable ethical standards. This study was performed in line with the principles of the Declaration of Helsinki. Approval was granted by the Ethics Committee of the Technical University of Munich.

## PATIENT CONSENT STATEMENT

Informed consent was obtained from all individual participants included in the study.

## Data Availability

The data that support the findings of this study are available on request from the corresponding author upon reasonable request.
